# Supplementary material for: Extreme purifying selection against point mutations in the human genome
Source: Nat Commun. 2022 Jul 25;13:4312. doi: 10.1038/s41467-022-31872-6 (PMC9314448; doi:10.1038/s41467-022-31872-6)
Supplement: Supplementary file 1 — Supplementary Information [file 41467_2022_31872_MOESM1_ESM.pdf]

Supplementary Information for: Extreme purifying selection against point mutations in the human genome

Noah Dukler[1,a], Mehreen R. Mughal[1,a], Ritika Ramani[1], Yi-Fei Huang[2], and Adam Siepel[1,*]

[1]Simons Center for Quantitative Biology, Cold Spring Harbor Laboratory, Cold Spring Harbor, NY
[2]Department of Biology and Huck Institutes of the Life Sciences, The Pennsylvania State University, University Park, PA
[a]These authors contributed equally to this work.
[*]Corresponding author: asiepel@cshl.edu

Supplementary Table 1: Ultraselection across the human genome (less conservative estimates)

Feature	λ_s	$\pm$ (stderr)	no. sites (M)	prop. sites	exp no. (M)[a]	exp. prop.[b]	fold enrich.	exp. s-del.[c]	s_{het}
CDS	0.148	0.0004	33.8	1.18%	4.9	23.3%	19.8	0.12	-
$5'$ UTR	-0.161	0.0006	8.2	0.29%	0.0	0.0%	0.0	0.00	-
$3'$ UTR	0.028	0.0002	36.1	1.26%	0.9	4.3%	3.4	0.02	-
splice	0.464	0.0012	0.8	0.03%	0.4	1.7%	63.0	0.01	2.0%
nonconserved lncRNA[d]	0.009	0.0001	453.6	15.78%	2.7	12.7%	0.8	0.06	-
conserved lncRNA[e]	0.055	0.0003	23.3	0.81%	1.2	5.7%	7.1	0.03	-
nonconserved intron[d]	0.009	0.0000	972.6	33.83%	6.4	30.3%	0.9	0.15	-
conserved intron[e]	0.058	0.0002	44.3	1.54%	2.5	11.7%	7.6	0.06	-
nonconserved intergenic[d]	0.003	0.0000	1255.5	43.67%	0.0	0.0%	0.0	0.00	-
conserved intergenic[e]	0.048	0.0002	46.9	1.63%	2.1	10.2%	6.2	0.05	-
Total			2875.1	100.00%	21.0	100.0%		0.51	

[a]Expected number of ultraselected sites after adjusting for background. In this case, the estimate for nonconserved intergenic regions (0.003) was subtracted from each estimate of λ_s (see **Table 1** for a more conservative correction).

[b]Expected proportion of ultraselected sites after adjusting for background.

[c]Expected number of new strongly deleterious mutations per diploid individual, assuming a mutation rate of 1.2×10^{-8} per generation per site.

[d]Sites not classified as conserved by phastCons.

[e]Sites classified as conserved by phastCons.

Supplementary Table 2: Means of full simulated DFE ($f(x)$), DFE associated with remaining rare variants ($g(x)$), and DFE inferred to be associated with the "missing" rare variants ($h(x)$) by mixture decomposition (see **Methods**). Also shown are the estimated values of λ_s from simulated data.

Distribution	α^a	θ^a	${\pi_0}^b$	mean $g(x)$	mean $f(x)$	mean $h(x)$	λ_s
Kim et al.,	0.1990	0.0331	3.1%	0.0039	0.0062	0.0326	0.0816
0d CDS	0.7500	0.0331	3.1%	0.0153	0.0240	0.0483	0.267
miRNA	0.9900	0.0331	0.0%	0.0206	0.0328	0.0552	0.354
TFBS	0.4500	0.0331	70.0%	0.0024	0.0045	0.0428	0.035

[a] Parameters of assumed Gamma distribution, where α is the shape parameter and θ is the scale parameter

[b] Weight of point mass at zero.

Supplementary Figures

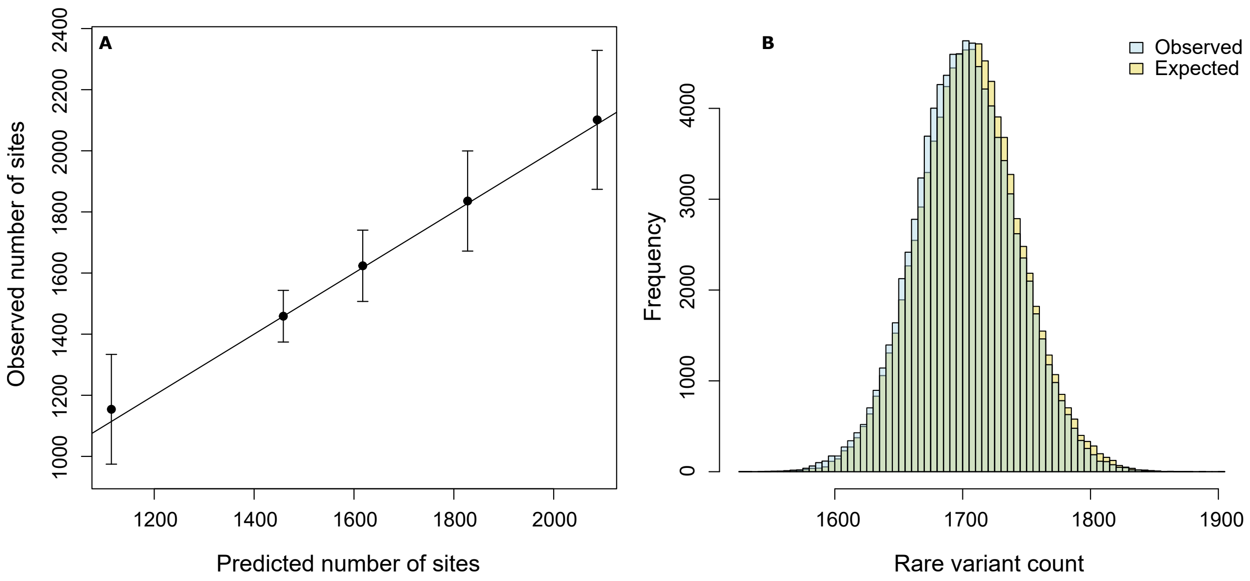

Supplementary Figure 1: **Predicted vs. observed numbers of rare variants in designated neutral regions.** (**A**) Predicted vs. observed mean numbers of rare variants in collections of 10,000 randomly sampled neutral sites. The total number of collections was 92,403 after filtering to eliminate repetitive sequences. Collections with similar numbers of predicted rare variants were grouped together, and then the mean prediction (x-axis) was plotted against the mean observation (y-axis; error bars represent one standard deviation) for each group. Groups were defined by intervals of 250 expected variants, i.e., 1000–1250, 1250–1500, . . . , 2000–2250 expected rare variants. (**B**) Full distributions of rare variant counts for the same 92,403 collections of 10,000 randomly sampled neutral sites, as predicted by our Bernoulli model (expected) and observed in the raw data (observed). The model-based distribution is obtained by sampling a rare variant with probability P_i at each site and then summing the rare variants across the 10,000 sites. One such sample was drawn for each of the 92,403 collections.

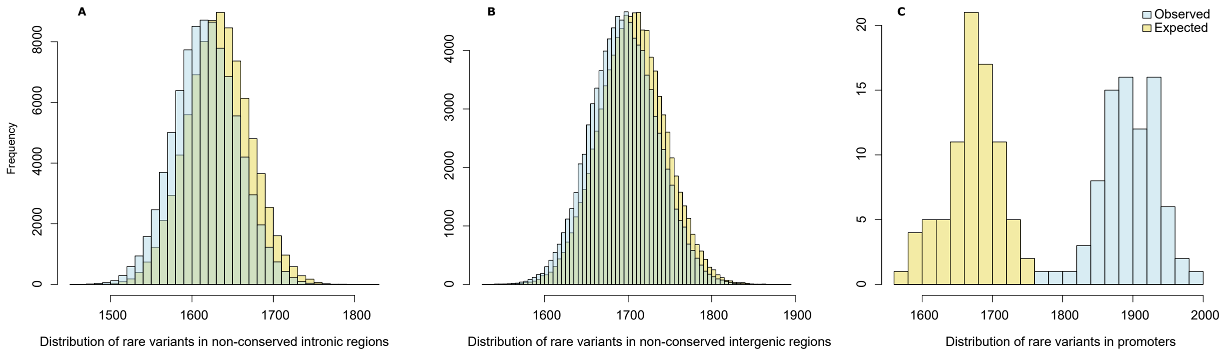

Supplementary Figure 2: **Distributions of observed vs. expected numbers of rare variants in other genomic regions.** Distributions are as described in Supplementary Fig. 1 but instead of designated neutral sites, we show results for (A) nonconserved (excluding phastCons elements) intronic regions, (B) nonconserved intergenic regions, and (C) promoter regions. Notice that the distributions match fairly well in (A) and (B) except for a slight downward shift in the observed data owing to low levels of ultraselection at unannotated elements; however, the promoters (C) display a pronounced shift toward excesses of rare variants not predicted by the model.

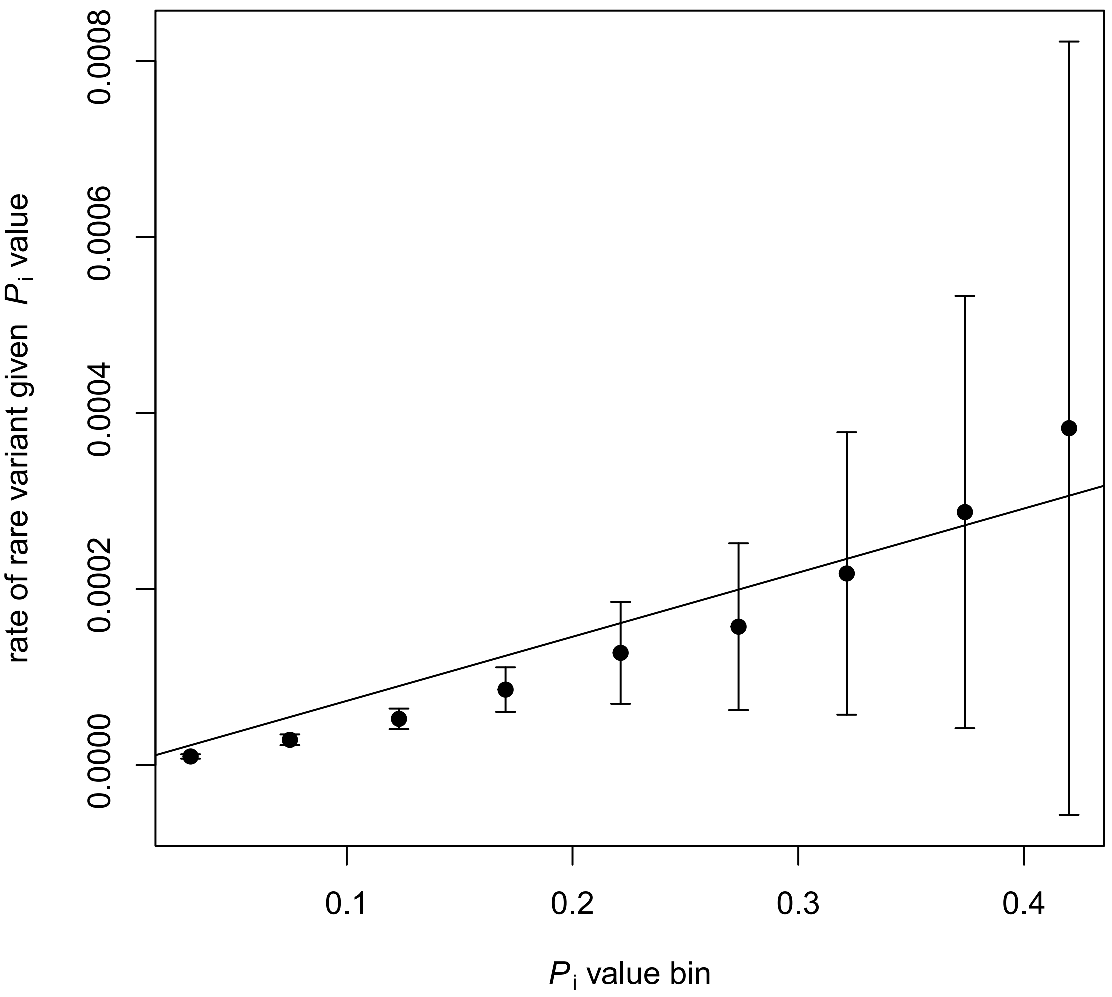

Supplementary Figure 3: **Predicted vs. observed rate of *de novo* variant sites.** A total of 174,122 *de novo* mutations from ref. [1] were grouped into bins by predicted P_i value (x axis) and plotted vs. the empirical rate at which variants occur within each bin (y axis). Rare variant counts are binned in intervals of 0.05 beginning at 0.00 and ending at 0.45. The number of rare variants in bins range from 483 variants to 47,523 variants. The line represents the least-squares fit for the mean values per bin. The error bars represent one standard-error in each direction according to binomial sampling.

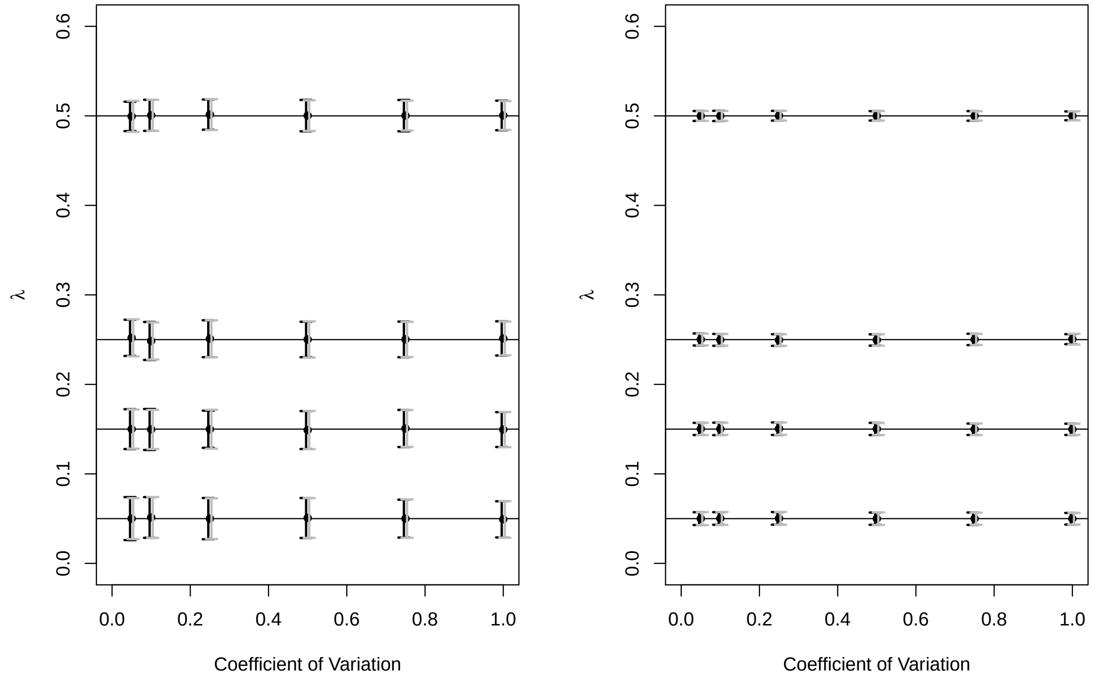

Supplementary Figure 4: **Simulations demonstrating that the estimates of λ_s are unbiased and have approximately the predicted variance.** Simulated data sets consisting of $M = 10,000$ (left) and $M = 100,000$ (right) sites were generated under the assumed model, with assumed values of $\lambda_s \in \{0.05, 0.15, 0.25, 0.50\}$. For each simulated data set, sitewise "true" values of P_i (indicating the rate at which neutral variants occur) were drawn from a normal distribution with mean $\mu = 0.15$ and standard deviation $\sigma = 0.068$, approximately as observed in our real data set. Estimated values of P_i were then drawn from a normal distribution with mean equal to the true P_i and increasing standard deviations, which are expressed as coefficients of variation relative to $\mu = 0.15$, as indicated on the x axis. Thus, the values of P_i used to estimate λ_s are unbiased but have increasing uncertainty from left to right in each plot. For each combination of a true λ_s and a coefficient of variation, a point is shown indicating the mean of the estimators for λ_s (based on equation 5) from 1000 replicated data sets (each with different P_i values). In addition, two sets of error bars are shown indicating the empirical standard deviation of these estimates (black) and the predicted standard error from equation 11 (based on the mean values of T, $\bar{P}$, and $\hat{\lambda}_s$ across replicates) assuming no error in the P_i values (gray). The solid horizontal lines indicate the assumed true values of λ_s. Notice that the estimates are unbiased with relatively small standard error in all cases. Notice also that the theoretical predictions of the variance (gray) almost perfectly match the empirical observations (black) despite that they ignore uncertainty in the P_i values.

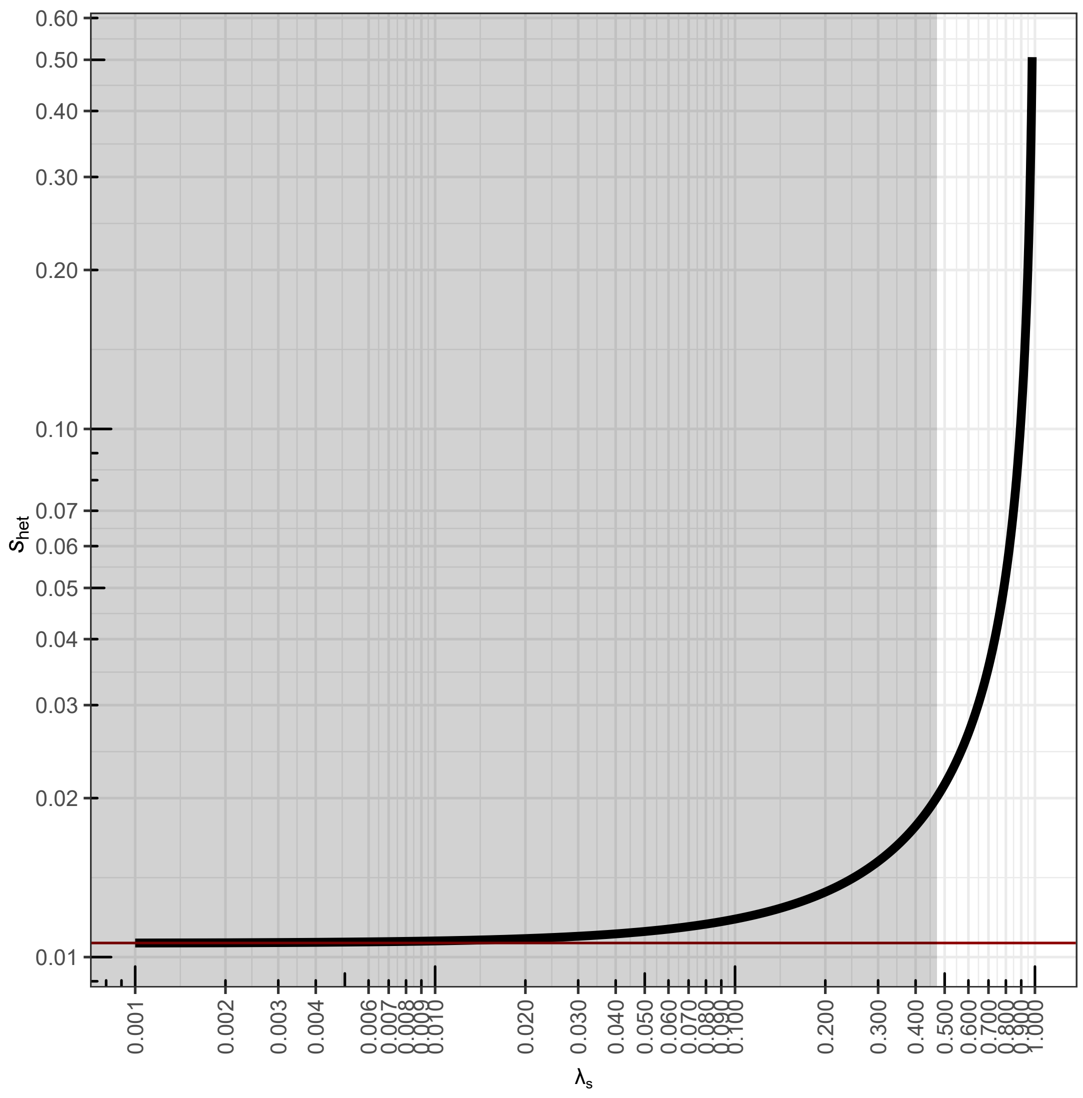

Supplementary Figure 5: **Theoretical relationship between λ_s and the selection coefficient against heterozygous mutations, s_{het}.** Curve represents equation 12 with $N = 71,702$ and $c = 1.35 \times 10^7$ based on our real data set (see **Methods**). The shaded region ($\lambda_s < 0.45$, $s_{\text{het}} < 0.02$) indicates the approximate regime where the relationship no longer yields an accurate estimator for s_{het} with our data (see **Supplementary Fig. 6**).

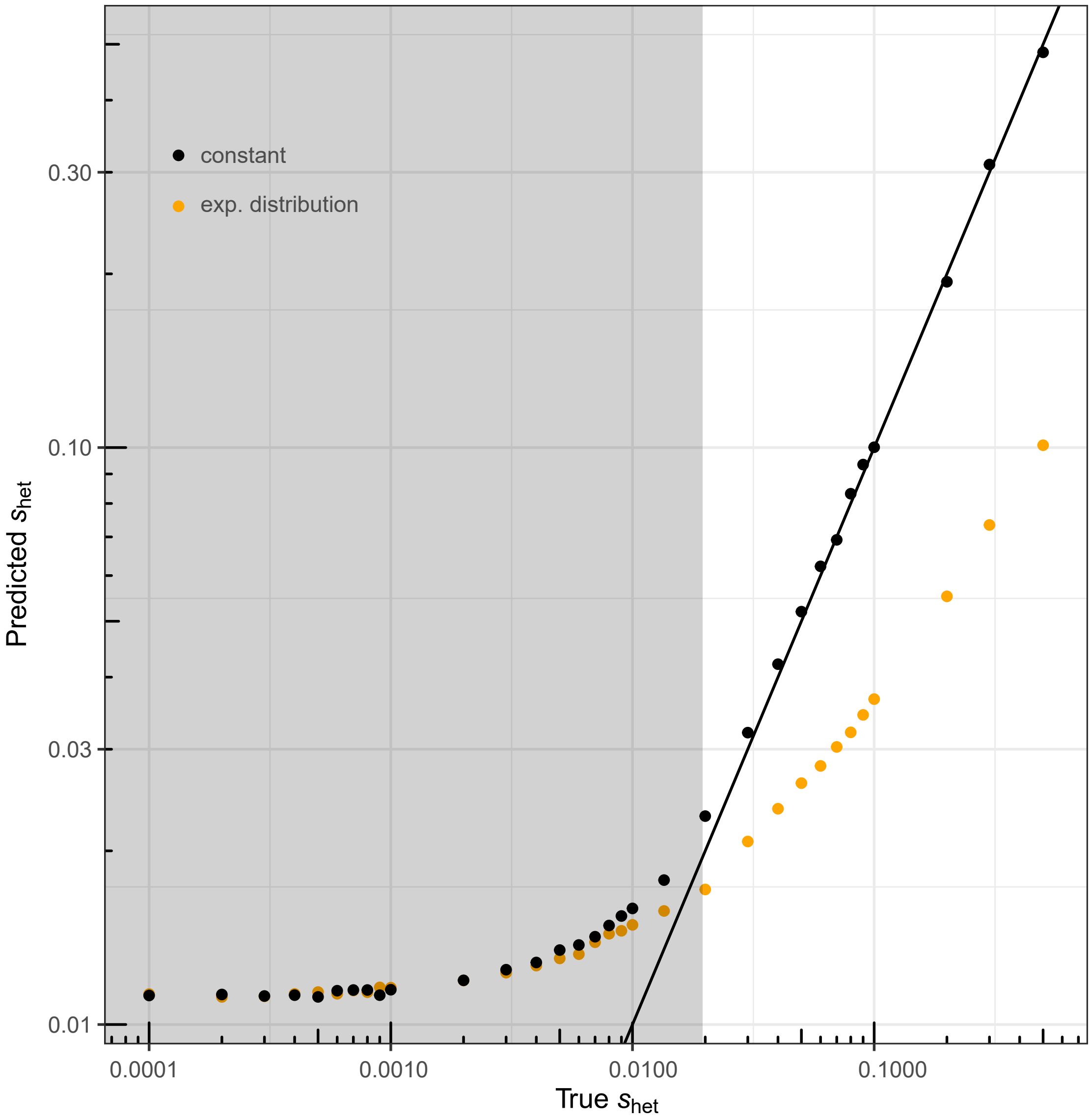

Supplementary Figure 6: **True vs. predicted values of s_{het} in simulation.** Data sets of 71,702 diploid individuals and 100,000 sites were simulated using software from ref. [2] with mean s_{het} ranging from 0.0001 to 0.5 (x-axis). In one version, all sites were assigned the same "true" value of s_{het} ("constant"; black points) and, in another, sitewise values of s_{het} were drawn from an exponential distribution with the given mean value ("exp. distribution"; orange points). ExtRaINSIGHT was applied to each simulated data set, and then the estimated value of λ_s was converted to a predicted s_{het} (y-axis) using equation 12. All simulations assumed a European demographic history (see **Methods**). As in **Supplementary Fig. 5**, the gray region respectively indicate the regimes in which the estimator for s_{het} is no longer useful.

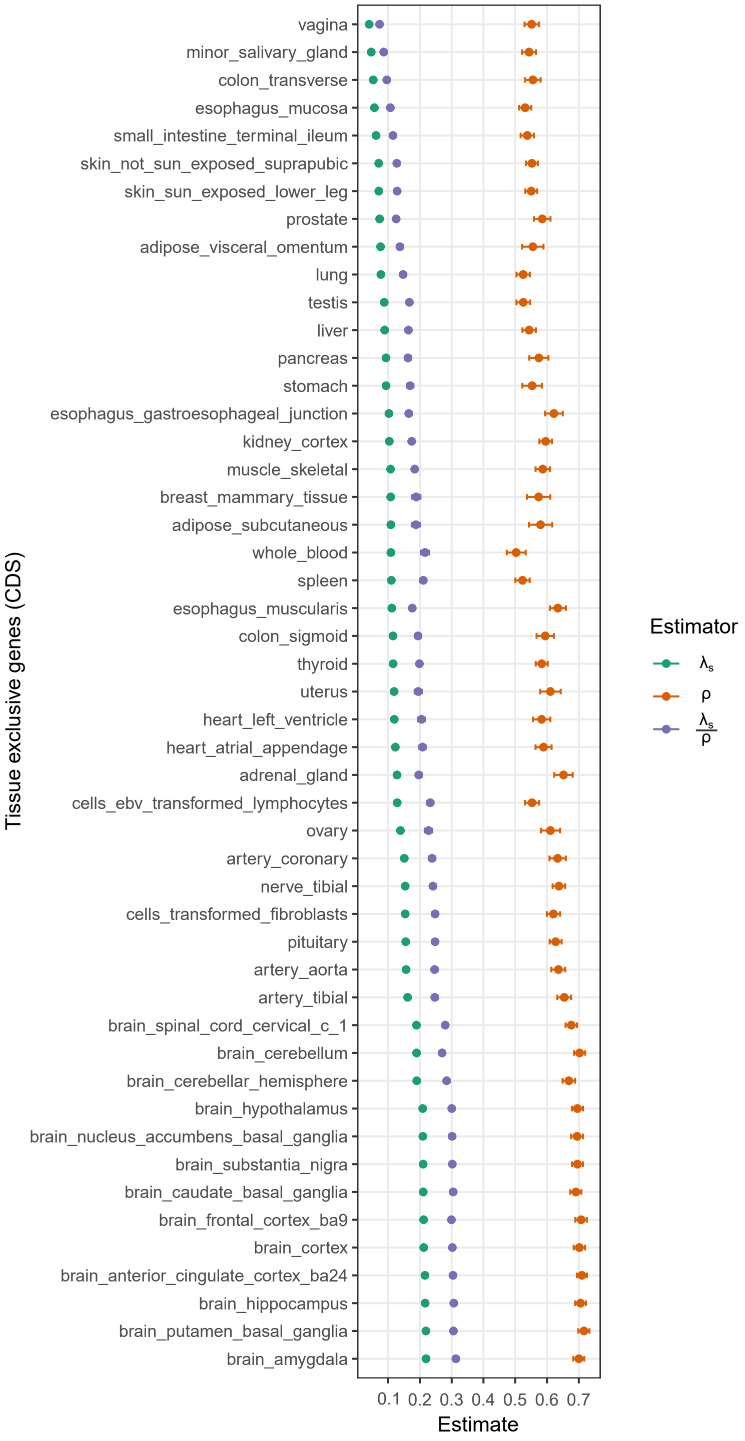

Supplementary Figure 7: **Measures of purifying selection in protein-coding genes exhibiting tissue-specific gene expression.** Tissue-specific genes were obtained from ref. [3] as detailed in the **Methods** section. An estimate for each tissue is shown for both ExtRaINSIGHT (λ_s) and INSIGHT (ρ). Error bars are centered at the MLE and indicate one standard error in each direction (see **Methods**). The number of genes per tissue ranges from 244–3,932.

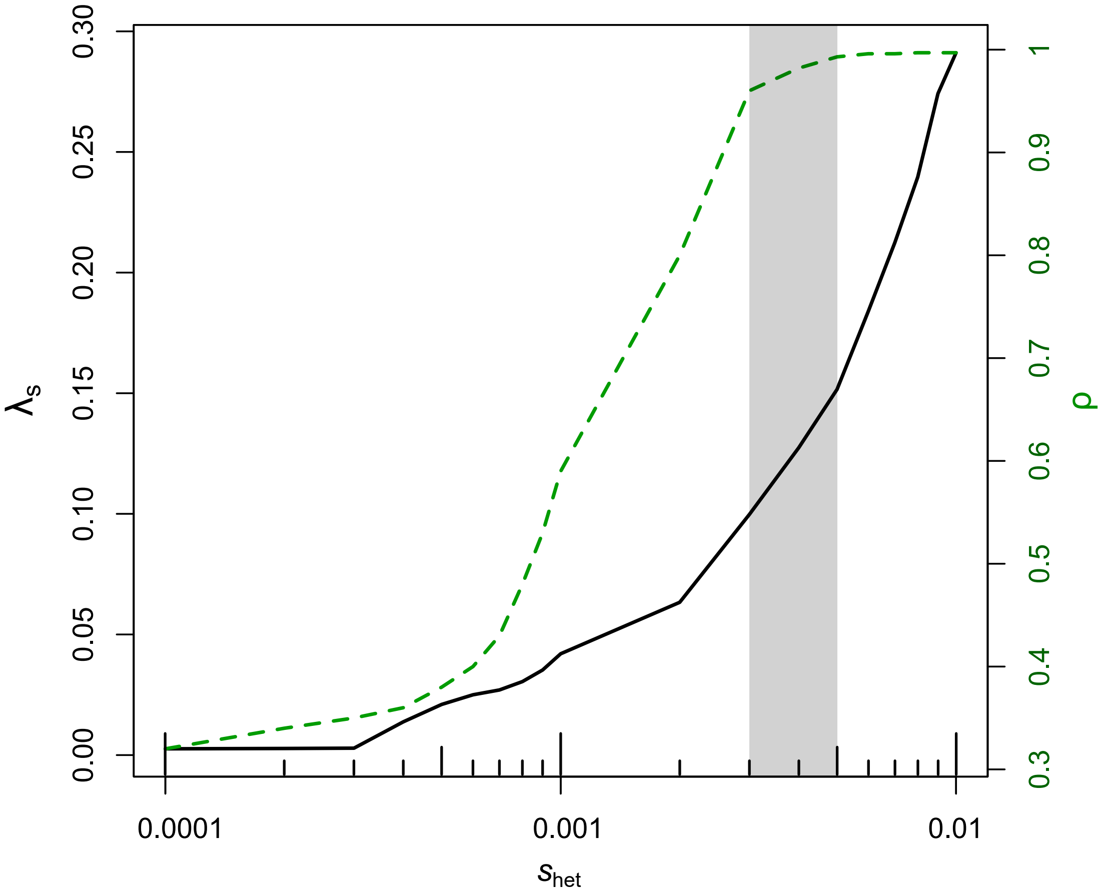

Supplementary Figure 8: **Measures of ultraselection and conservation at different strengths of s_{het}.** To measure ultraconservation (λ_s), we simulate 100,000 sites for 71,702 diploid individuals using software from ref. [2] with s_{het} ranging from 0.0001 to 0.01 (x-axis). To measure conservation (ρ), we simulate a 1MB region for chimpanzee and human populations with N_e of 20,000 and 10,000 respectively with constant demographic history using SLiM [4] with s_{het} again ranging from 0.0001 to 0.01. We compare the divergence in these simulations to divergence under neutrality, where divergence is measured by the number of sites in one sampled human chromosome that differ from one sampled chimpanzee chromosome. ρ is computed as $1 - \frac{\text{div}_{sel}}{\text{div}_{neut}}$, where div_{sel} is divergence in simulations of selection and div_{neut} is divergence in neutrality.

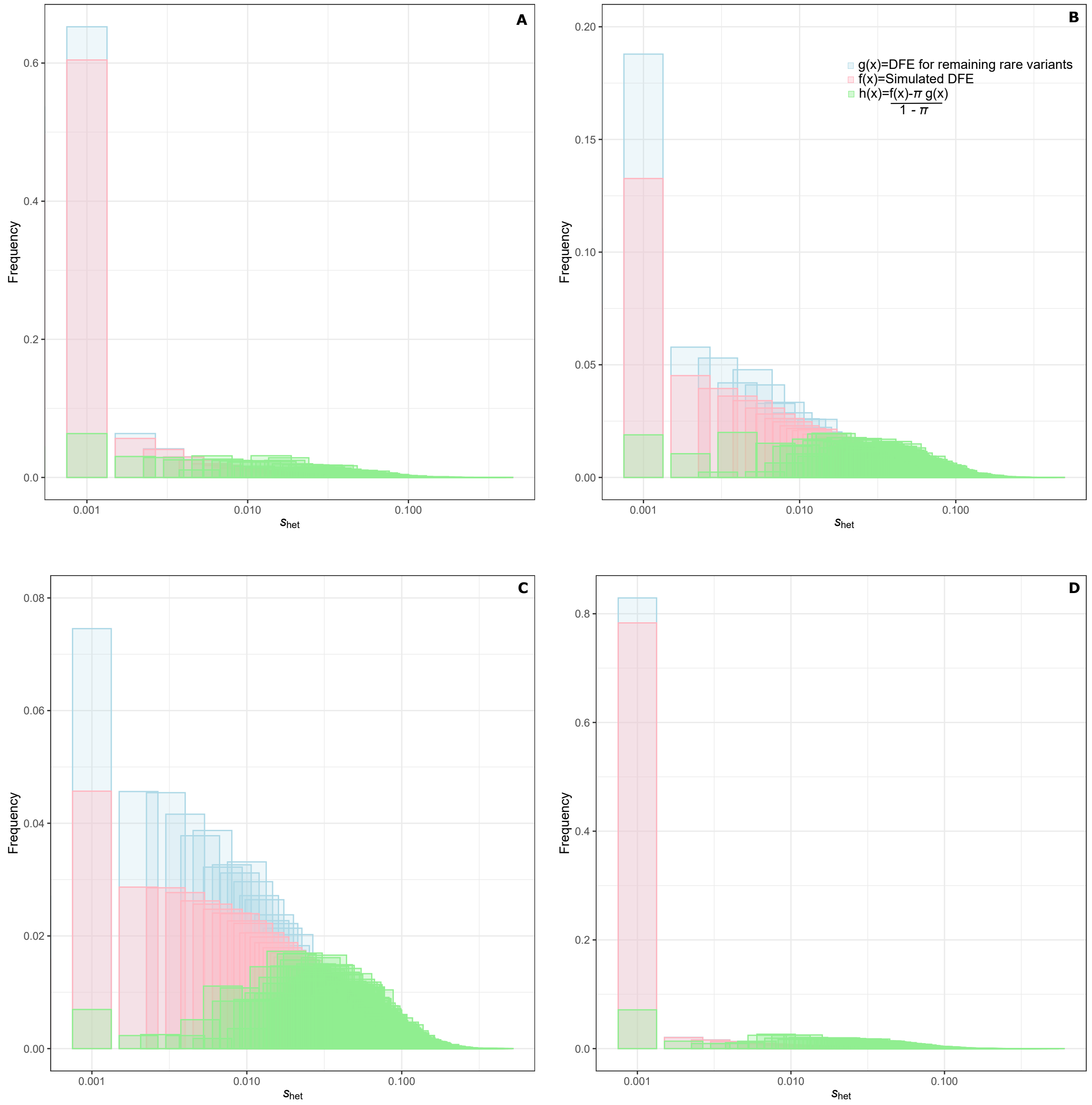

Supplementary Figure 9: **Comparison of DFEs for all sites, rare variants that remain, and "missing" rare variants in simulations.** Simulated DFEs ($f(x)$; pink), DFEs for rare variants that remain in the data ($g(x)$; blue), and DFEs inferred by mixture decomposition for the rare variants that are missing ($h(x)$; green). Results are shown for four distinct DFEs: **(A)** a DFE published by Kim et al. [5] consisting of a mixture of a point-mass at zero (with weight 0.031) and a Gamma distribution with α=0.1990 and θ=0.0331. **(B)** a modified DFE designed to approximately match our observations at 0d sites in coding regions, consisting of a mixture of a point-mass at zero (weight 0.031) and a Gamma distribution with α=0.75 and θ=0.0331. **(C)** a modified DFE designed to approximately match our observations at evolutionarily ancient miRNAs, equal to a Gamma distribution with α=0.99 and θ=0.0331. **(D)** a modified DFE designed to approximately match our observations at TFBS, consisting of a mixture of a point-mass at zero (with weight 70%) and a Gamma distribution with α=0.45 and θ=0.0331. Means of these distributions along with our λ_s estimates are shown in **Supplementary Table 2.**

Supplementary References

[1] Turner, T. N. *et al.* denovo-db: a compendium of human *de novo* variants. *Nucleic Acids Research* **45**, D804–D811 (2016).

[2] Weghorn, D. *et al.* Applicability of the mutation-selection balance model to population genetics of heterozygous protein-truncating variants in humans. *Mol Biol Evol* **36**, 1701–1710 (2019).

[3] Yang, R. Y. *et al.* A systematic survey of human tissue-specific gene expression and splicing reveals new opportunities for therapeutic target identification and evaluation. *bioRxiv* (2018).

[4] Haller, B. C. & Messer, P. W. SLiM 2: flexible, interactive forward genetic simulations. *Molecular Biology and Evolution* **34**, 230–240 (2017).

[5] Kim, B. Y., Huber, C. D. & Lohmueller, K. E. Inference of the distribution of selection coefficients for new nonsynonymous mutations using large samples. *Genetics* **206**, 345–361 (2017).
